# Short Mindfulness-Based Relaxation Training Has No Effects on Executive Functions but May Reduce Baseline Cortisol Levels of Boys in First Grade: A Pilot Study

**DOI:** 10.3390/children9020203

**Published:** 2022-02-04

**Authors:** Adam Koncz, Reka Kassai, Zsolt Demetrovics, Zsofia K. Takacs

**Affiliations:** 1 Doctoral School of Psychology, ELTE Eötvös Loránd University, 1064 Budapest, Hungary; koncz.adam@ppk.elte.hu (A.K.); kassai.reka@ppk.elte.hu (R.K.); 2 Institute of Psychology, ELTE Eötvös Loránd University, 1064 Budapest, Hungary; demetrovics@t-online.hu; 3 MTA-ELTE Lendület Adaptation Research Group, The Hungarian Academy of Sciences, 1064 Budapest, Hungary; 4 Institute of Health Promotion and Sport Sciences, ELTE Eötvös Loránd University, 1117 Budapest, Hungary; 5 Centre of Excellence in Responsible Gaming, University of Gibraltar, Gibraltar GX11 1AA, UK; 6 School of Health in Social Science, University of Edinburgh, Edinburgh EH8 9AG, UK

**Keywords:** mindfulness, school entry, executive functions, stress, intervention

## Abstract

(1) Background: Executive functions are important for academic performance and school readiness. Children’s executive function skills are found to be improved by mindfulness-based interventions, and these programs are also effective in stress reduction. The aim of this study was to evaluate the feasibility and the effects of a short mindfulness-based relaxation training compared to a passive control condition right before school entry on executive function skills and cortisol levels. (2) Methods: The feasibility and the effects of the intervention before school entry were tested with 61 preschoolers. The final sample consisted of 51 participants (M_age_ = 81.90 months, *SD* = 5.45; 41% male). Short-term memory, executive function skills and cortisol levels before and after the intervention were assessed. Additionally, cortisol levels were assessed one week and one month after school entry. (3) Results: There was a significant sex difference in the effects of the intervention on children’s cortisol levels (*p* = 0.026, *η^2^* = 0.134). The mindfulness-based relaxation training applied before school entry prevented a rise in boys’ cortisol levels one week after starting school. (4) Conclusion: A short mindfulness-based intervention before starting school could be effective in fostering physiological stress management in boys.

## 1. Introduction

### 1.1. Background and Condition

An emerging number of studies show that self-regulatory skills and executive functions play an important role in school readiness and academic performance [1,2], even more so than IQ [3]. Executive function skills are usually considered to consist of three distinct components: inhibition, working memory and cognitive flexibility [4,5,6]. These skills allow us to organize our behavior according to goals instead of acting automatically. Inhibitory control is the ability to suppress a response that is not appropriate in a situation [6], working memory is where information is being temporary stored, until transferred to long-term memory or processed, and shifting or cognitive flexibility is the ability to flexibly change between different rules. For fostering children’s executive functions, several possibilities have been proposed, such as computer programs, curricula, yoga, mindfulness meditation and sports [5]. In a meta-analysis, Takacs and Kassai [7] found mindfulness-based interventions one of the most effective intervention methods for typically developing children for enhancing working memory and inhibition skills, although the number of studies was limited. Additionally, Moore and Malinowski [8] found higher performance on a cognitive flexibility test among meditators. In sum, all three components of executive function might be improved with practicing meditation.

Practicing mindfulness means focusing on sensations and thoughts in the present moment in a non-judgmental way [9]. The efficacy of mindfulness-based programs for children’s executive function skills might be explained by the notion that self-regulation skills in early childhood are affected by controlled processes such as executive functions and automatic processes like stress and anxiety [10]. Thus, an ideal intervention might target both, ensuring children exercise cognitive functions (e.g., executive functions) and aiding stress management. Mindfulness practices require the meditator to focus on the sensations or thoughts in the present moment and redirect their attention back to this target when their mind wanders. Thus, practicing mindfulness demands conscious monitoring of one’s attention. Additionally, mindfulness-based programs have been shown to reduce anxiety and both perceived [11] and physiological stress [12,13,14]. The combination of the two kinds of processes might make mindfulness-based interventions especially effective in fostering children’s self-regulatory and executive function skills [10].

Physiological stress response is often measured by the product of the operation of the hypothalamic–pituitary–adrenal axis: the hormone cortisol, which is regarded as an objective stress biomarker [15]. It can be assessed in non-invasive manners, such as from saliva samples. Cortisol levels in saliva samples are correlated with serum levels [16]. The production of this hormone has a circadian rhythm: levels of salivary cortisol are higher in early morning and decrease throughout the day until midnight [17]. Baseline cortisol levels can provide information about chronic physiological stress. For instance, Lindholm and colleagues [18] found higher salivary cortisol after awakening in those who had stressful jobs. Cortisol levels also elevate temporarily in the case of acute stress in healthy individuals. This elevation is highest in social-evaluative and uncontrollable situations, such as the Trier Social Stress Test [19]. Physiological stress reactivity is also assessed with children with an adopted version of the Trier Social Stress test [20].

Results regarding sex differences in baseline cortisol levels are mixed. Kirschbaum, Wüst and Hellhammer [21] failed to find any effect of sex on baseline salivary cortisol levels in three studies. In contrast, Larsson and colleagues [22] showed higher morning baseline blood cortisol levels in women, while Roelfsema and colleagues [23] found lower total daily blood cortisol production in premenopausal women compared to men.

There is no clear evidence for a sex difference in physiological stress reactivity in healthy children [24]. However, Mazurka and colleagues [25] found among depressed adolescents that boys show higher cortisol reactivity than girls. In the case of adults, women were found to produce less cortisol in reaction to stress [26,27].

Protection from chronic stress (e.g., stress caused by low socioeconomic status) in childhood is important because it can lead to abnormal brain development and decreased cognitive functioning [28]. Elevated cortisol levels or problems in cortisol regulation have been linked to not only familiar or household problems like poverty [29] or maltreatment [30], but one study found higher baseline cortisol levels in children in childcare [31]. Interestingly, Groeneveld and colleagues [32] found elevated cortisol levels after school entry, which suggests that starting school might be a stressful life event.

Acute increase of cortisol in response to physically and/or mentally challenging situations is not necessarily a reaction that should be eliminated. It simply reflects a reaction to a situation that requires adaptation. In fact, physiological stress response in the normal domain is adaptive in demanding situations [33] or in changing environments [34]; under such circumstances, the lack of a stress response might be more problematic. For example, cortisol plays a role in setting the optimal arousal level. Performance of prefrontal functions (such as executive functions) improves with arousal until a certain point, but then it starts to decrease [35]. A similar inverted u-shaped pattern has been shown in the case of the flow experience [36], and between sympathetic arousal and cortisol levels [37]. Consequently, optimal stress levels are important in order to maintain cognitive performance [38,39,40,41]. During chronic stress, however, cortisol production of the HPA-axis could become atypical, i.e., it does not necessarily imply a large increase in response to a stressful situation; rather, a blunted cortisol reactivity or prolonged increased levels without recovery can be observed [42].

The effects of meditative programs on cortisol levels have been investigated in meta-analyses. Pascoe and colleagues [12] found a significant medium-sized effect of meditation on blood cortisol, while Sanada and colleagues [13] found a moderate-sized effect of mindfulness programs on salivary cortisol levels. Although meta-analytic evidence suggests that mindfulness programs are effective in reducing adults’ baseline cortisol levels, there is very limited evidence regarding children [14]. Moreover, in this meta-analysis we found larger effects of meditative interventions on blood cortisol samples in trials involving more male participants. Thus, there is some evidence that mindfulness-based interventions might be more effective in reducing males’ blood cortisol levels. Sibinga and colleagues [43] tested the effects of a 12-session-long mindfulness-based stress reduction (MBSR) program compared to a health education program among seventh- and eighth-grade boys from low-income families. Results show that participants of the MBSR program benefited; their salivary cortisol levels increased to a lesser extent than those of the control group. In contrast, Schonert-Reichl and colleagues [44] tested the effect of a mindfulness-based social emotional learning (SEL) program among fourth- and fifth-graders and found that morning salivary cortisol levels were higher on post- but not on pre-tests in those children who attended the 12-lesson-long SEL program compared to those who participated in a social responsibility program.

The effects of mindfulness on cortisol reactivity are controversial. Brown et al. [45] found that trait mindfulness is associated with lower cortisol reactivity to a TSST task among adults. On the other hand, a short (three-session-long) mindfulness program was found to increase salivary cortisol reaction compared to a cognitive training program [46].

In sum, mindfulness-based interventions seem to have positive effects on executive function skills and both psychological and physiological stress; however, evidence regarding children is limited. Furthermore, such programs might have the potential to foster children’s adaptation and performance in stressful life situations, such as school entry; however, no studies to our knowledge have tested this.

### 1.2. Motivation and Goals

On the basis of the above-mentioned literature, the present study aimed to test whether a short mindfulness-based relaxation training session is feasible right before school entry. We also tested whether this intervention could improve executive function skills and lower children’s morning physiological stress levels (measured by cortisol) upon school entry as well as their stress/cortisol reactivity.

In line with the above-presented findings, we hypothesized that (i) a short mindfulness-based program enhances children’s executive function skills, especially working memory and inhibitory control. Furthermore, we hypothesized that (ii) the mindfulness program will decrease children’s morning cortisol levels compared to the control group. We also hypothesized that (iii) the mindfulness intervention will protect children from elevated cortisol levels upon school entry on follow-up assessment, and that (iv) the mindfulness group will show lower cortisol reactivity to an acute stress situation post-test.

## 2. Materials and Methods

### 2.1. Participants

Preschoolers facing school entry were recruited in eight state preschools in the capital of Hungary, Budapest, during the summer of 2017 and 2018. Of the approximately 90 consent forms distributed one week before the pre-test session, 62 parents indicated their child’s willingness to participate. The inclusion criterion was the child was not having any psychological or psychiatric problems or any somatic illnesses that could have an impact on cortisol levels (e.g., type II diabetes). Five children had to be excluded for a variety of reasons (not native speaker of Hungarian (*n* = 1), refused to participate in the pre-test session (*n* = 1), was absent during the pre-test week (*n* = 1). Additionally, we also had to exclude one member each of two pairs of twins because these children could not be considered independent participants (*n* = 2). Finally, 57 children (92%) were randomized to either the control (*n* = 28) or the intervention group (*n* = 29). Since the study was conducted in August, some children dropped out of the experiment because they were absent from preschool, as they went on holiday with their family. A total of 26 of the 28 children in the control group (93%) were in the kindergarten during the intervention period (two children went on holiday). Three intervention participants dropped out during the intervention because they went on holiday; thus, 26 of the 29 children in the intervention condition participated in more than half of the intervention sessions (10% dropout), one of the 26 children participated on only 3 sessions, while others attended all sessions. Additionally, one control participant decided during the post-tests not to participate further in the study. The final sample consisted of 51 participants aged 71 to 94 months (*M* = 81.90, *SD* = 5.45). In addition, 41% of the sample was male.

**Figure 1:**
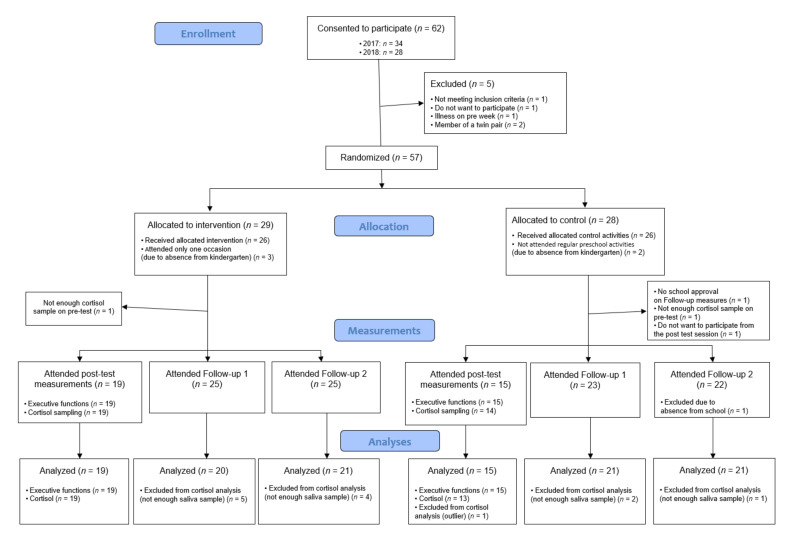
CONSORT diagram: Number of participants in the control and intervention group at each timepoint.

The socioeconomic status of the participants was most likely close to the average of Budapest, the capital of Hungary, because all the participants were recruited from state preschools from average SES districts of Budapest. However, we had insufficient data to draw a firm conclusion, as most parents did not fill in the demographic questionnaire. For the exact number of participants whose data could be included in each analysis, see Figure 1, and for descriptive details of the intervention and control groups, see Table 1 and Table 2.

### 2.2. Design

**Figure 2:**
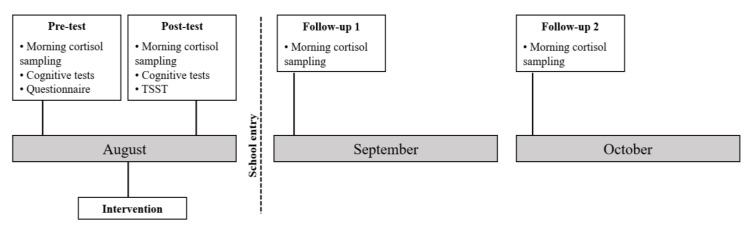
Timeline of the experiment.

A pilot study with a between-subject design was applied. Participants in the different preschool classrooms were matched based on age, sex and pre-test executive function performance and randomly assigned to the experimental or the passive control group. On a week in August, preceding school entry in September, the experimental group was taken out of the classroom for five 30 min sessions of mindfulness-based relaxation training, while the passive control group attended regular preschool activities, which consisted of free play at the times of the intervention sessions. Measurements were implemented at four time points: on the week before (pre-test) and the week after (post-test) the intervention in August in kindergarten. Additionally, there were two follow-up (FU) times: the first week of school (first week of September (FU1)) and one month after school entry (the first week of October (FU2)). There was cortisol sampling in the morning upon arrival to the preschool at all four measurement time points and an individual session including executive function tests at pre- and post-test times. For the executive function tests, different stimuli were used for the pre- and post-test in order to avoid a learning effect. Additionally, we applied the Trier Social Stress Test adapted for children (TSST-C) on the post-test, following the executive function tests. On follow-up, there was only cortisol sampling in the morning upon arrival to school (see Figure 2). Due to funding restraints, one cortisol sample per measurement occasion was taken from the children in the sample recruited in 2017, and one sample on three consecutive weekdays on each measurement occasion was taken from the children recruited in 2018. These samples were taken upon arrival at the institution on the mornings of Tuesdays, Wednesdays and Thursdays in order to avoid effects of the beginning or the end of the (pre-)school week.

### 2.3. Procedure

Recruitment was done through the children’s kindergarten teachers. We contacted the head of the kindergarten, who offered to talk to the kindergarten teachers about the possibility of taking part in the study. The kindergarten teachers distributed and collected the consent forms from the parents. As all elements of the data collection took place in educational institutions (kindergartens and schools), we then contacted the head teachers of the schools where the children started their studies in September.

Morning cortisol samples were taken upon arrival at the preschool and school. Because children did not arrive at exactly the same time, we registered the time of sampling. To check the possible differences in the time of sampling (the one recorded value was used in case of data collected in 2017 and the average of the three days was used in case of the data from 2018) between the control and intervention group or between boys and girls, we ran repeated measures ANOVAs. Measurement points (e.g., pre-test, post-test, follow-up 1, follow-up 2) were used as the within-subject factor, while sex and condition were the between-subject factors. For children whose data could be included in the analyses regarding the pre- to post-test change in baseline cortisol (*n* = 33), the time of the sampling did not differ between the pre- (*M* = 8:33, *SD* = 0:35) and the post-test (*M* = 8:44, *SD* = 0:34), and there was also no main effect of condition or sex. There was also no interaction effect between time × condition, time × sex or time × condition × sex (for test statistics, see Appendix A).

For children whose data could be included in the analysis regarding the change from pre-test to September (*n* = 41), samples were taken significantly earlier at the follow-up (at school) (*M* = 7:44, *SD* = 0:10) than for the pre-test (in preschool) (*M* = 8:38, *SD* = 0:35). This is conceivable, as school starts at 8:00 AM in most schools in Hungary, while there is more flexibility in when children should arrive in preschool in the summer. There was no main effect of condition or sex, or any interaction effects between time × condition, time × sex, or time × condition × sex (for test statistics, see Appendix A).

Similarly, for children whose data could be included in the analysis regarding the change from pre-test to October (*n* = 42), the sampling time was also significantly earlier at school (*M* = 7:46, *SD* = 0:09) than in preschool (*M* = 8:39, *SD* = 0:36). There was no main effect of sex or condition or any interaction between time × sex, time × condition, sex × condition or time × sex × condition (for test statistics, see Appendix A).

The executive function tests were implemented by research assistants in a quiet room in the kindergarten. Every session was recorded on a camcorder for the non-digitized tests to be coded by two independent coders afterwards. Disagreements were settled in discussion. The Go/No-Go test results were recorded by PsychoPy (ver. 1.85.1) [47]. At the beginning of the testing session, children received a certificate for which they could choose stickers after completing each task, as a means of motivation.

### 2.4. Intervention Materials

The experimental group attended a one-week (5 sessions) story-based mindfulness-based relaxation program in the kindergarten in small groups (2–5 children). Every session was about 25–30 min long. The intervention was compiled by the authors based on commercially available books on mindfulness practices and relaxation storybooks for children. Similar to other mindfulness-based interventions in the literature, the program was quite complex and included a wide range of exercises: breathing and sensory meditations, progressive muscle relaxation and yoga postures. For more details and references, see Appendix B.

### 2.5. Measurement Instruments

#### 2.5.1. Baseline Cortisol Levels

Sample collection was implemented immediately after participants arrived to preschool or school in the morning. This was done on three consecutive weekdays in 2018 but only on one weekday in 2017 at each time point (pre- and post-test and the two follow-up occasions). In order to measure cortisol reactivity, we also took saliva samples before and after the TSST-C procedure (for details, see below).

Saliva samples were collected using children’s swabs (Nobaophtalm, Ko-Medic, Debrecen, Hungary). Unstimulated saliva samples were collected for 1–2 min, and then swabs were placed into sterile Eppendorf tubes and stored at −20 °C until assay. Free cortisol in saliva samples was measured by ELISA research kits (NT-DSNOV20, Novatec GmBH, Hattersheim am Main, Germany) designed for salivary samples, according to the manufacturer’s instructions. The intra- and interassay variations (CV%) were 3.5 and 9.2%, respectively. All samples from the same person were measured on the same plate.

#### 2.5.2. Stress Reactivity

A stress induction task, the Trier Social Stress Test for Children, which is a modified version of Trier Social Stress Test reported by Gilissen and colleagues [48], was used on the post-test immediately after the executive function tests. In the first part of the task, in order to simulate public speaking, the experimenter started a story and asked the child to think for two minutes and then finish that story in front of a camera. The experimenter told the child that there were people sitting behind the camera who would evaluate his/her speech. In the second part of the test, a puzzle was given to the child that was extremely difficult for this age group. The experimenter told the child that they had four minutes to solve it and that his/her peers could easily do it. After four minutes, the experimenter indicated that the time had expired and said that he/she was leaving the room to ask those people who are behind the camera about the child’s storytelling performance. When the experimenter came back, he/she told the child that he/she was just as good at storytelling as his/her peers and apologized for the mistake, namely that the puzzle was way too difficult.

To measure cortisol reactivity, five cortisol samples were collected from participants during this test. The first sample was taken upon arrival to the testing room before starting the executive function tests. A second sample was taken immediately after the stress test. Afterwards, children were taken to a separate room, where they could sit and color or play until all subsequent saliva samples were collected. The following three samples (after 15–30–45 min) were taken in that room. The area under the curve (AUC) during the stress test was calculated by the trapezoid formula presented by Pruessner and colleagues [49]. There are two types of data that can be computed: the area under the curve with respect to ground (AUCg) and the area under the curve with respect to increase (AUCi). The AUCg is informative regarding the overall cortisol production during the sampling procedure, while the AUCi contains information about the change as compared to the baseline during the procedure.

Three participants received the TSST test in the afternoon. As cortisol values and patterns can be very different before and after noon because of the diurnal rhythm of the cortisol production of the human body, the data of these participants was excluded from the analyses regarding cortisol reactivity.

#### 2.5.3. Short-Term and Working Memory

For measuring short-term memory and working memory, digit span forward and backward tests were used, respectively [50]. The items were recorded and played on a laptop in order to ensure a standardized presentation. There were two practice items before both the forward and backward parts, including two digits per item. On every level there were two items, but the number of the digits increased by one at each level. The test was finished when the child made a mistake on both items. For every correct item the child received one point.

#### 2.5.4. Inhibition and Cognitive Flexibility

We used a modified version of the fish and shark Go/No-Go task [51], in which the participant is asked to press a button for the go stimulus but not to press for the no-go stimulus. We used pictures of animals as stimuli (cat and tiger on the pre-test, fish and shark on the post-test). Each stimulus was presented for 1500 ms unless the participant responded by pressing a button. Before starting the task, six practice trials were presented. After this first block, we switched the rule so that children were asked not to respond to the go stimulus of the first block (e.g., the fish) and press the button for the no-go stimulus of the first block (e.g., the shark). In the third block, the rules changed back to the rules of the first block. These rule switching blocks were included as an attempt to assess cognitive flexibility. Thus, commission errors were calculated for the first block and considered as a measure of inhibitory control, and the sum of the commission errors in the second and third blocks was taken as an indicator of cognitive flexibility. Each block consisted of 16 go and 8 no-go trials. The test was run on PsychoPy 1.85.1 version [47].

However, results of this task showed floor effect (commission errors at pre-test in the intervention group were *M_block1_* = 0.15 (*SD* = 0.37), *M_block2_* = 0.42 (*SD* = 0.58), and *M_block3_* = 0.77 (*SD* = 0.81) and in the control group were *M_block1_* = 0.60 (*SD* = 1.63), *M_block2_* = 0.80 (*SD* = 1.70), and *M_block3_* = 0.64 (*SD* = 1.15); the change scores from pre- to post-test in the intervention group were *M_block1_* = −0.74 (*SD* = 1.05), *M_block2_* = −0.21 (*SD* = 0.92), and *M_block3_* = −0.32 (*SD* = 1.25) and in the control group were *M_block1_* = 0.00 (*SD* = 1.00), *M_block2_* = −0.38 (*SD* = 0.87), and *M_block3_* = −0.15 (*SD* = 1.07). Thus, we did not conduct analyses on these variables.

Cognitive flexibility. The Dimensional Change Card Sort (DCCS) task [52] was used to measure cognitive flexibility. We used green and yellow cars and flowers as the stimuli on the pre-test, while red and blue rabbits and boats were applied on the post-test. This task consisted of two parts. The first part was the standard version, in which the child was asked to sort cards based on one dimension: based on color in the first block and based on shape in the second block. There were two practice trials followed by six test trials in the first block. After that, the last six cards were sorted based on shape (no practice trials). In the second part of the test, cards with the same stimuli were used, except that half of the cards had a black border around them and half did not. Children were asked to sort the cards according to color or shape, depending on whether the card had a border or not. In this second part, there were two practice and 12 test trials. The number of correct trials on the third block was calculated.

#### 2.5.5. Behavior Problems

To assess behavior problems of the participating children, the short Hungarian version of the Child Behaviour Checklist [53] (translated by Gádoros [54]) was filled in by the parents before the intervention (pre-test in August) and one month after school entry (follow-up in October). This questionnaire consists of 46 items and six scales: Social Problems, Anxious/Depressed, Somatic Complaints, Attention Problems, Rule-Breaking Behaviour, Aggressive Behaviour.

From the total sample (*n* = 51), only parents of 16 children filled in the questionnaires at the pre-test and follow-up time points; thus, the results of these questionnaires could not be analyzed.

### 2.6. Statistical Analyses

We used the time of sampling during the day as a covariant in the cortisol analyses because there was quite some variation in when we could take samples in the morning or when we could start the stress induction test. ANOVAs were applied. The assumption of normal distribution was assessed by the standardized skewness and kurtosis values not exceeding +/− 1.96 [55].

## 3. Results

### 3.1. Feasibility

No children assigned to the intervention group refused to partake on any of the intervention sessions. Children only dropped out of the study at this phase due to absence from the preschool. They generally enjoyed the sessions and complied with the research assistants leading the sessions.

### 3.2. Cognitive Skills

#### 3.2.1. Pre-Test Differences

A total of 17 children did not attend the post-test session (7 in the intervention and 10 in the control group); thus, the final sample for these analyses consisted of 34 participants. For testing any possible differences at pre-test we ran a univariate ANOVA, with the scores on each test as the dependent variable and condition and sex as fixed factors. We did not find any main effects of condition or sex or a condition × sex interaction on the digit span forward, the digit span backward or the DCCS tasks (for descriptive statistics see Table 2, and for test statistics see Appendix C).

#### 3.2.2. Short-Term Memory

To test the effect of the intervention on the digit span forward task, a repeated measures ANOVA was applied, with time as the within-subjects factor (pre-test and post-test) and condition and sex as between-subjects factors. There was a significant main effect of time: children’s short-term memory performance decreased in the whole group from pre-test (*M* = 5.24, *SD* = 1.37) to post-test (*M* = 4.79, *SD* = 1.27). There were no main effects of condition or sex, and no significant time × condition, time × sex or time × condition × sex interactions (for descriptive statistics see Appendix D, and for test statistics see Appendix E).

#### 3.2.3. Working Memory

A Repeated Measures ANOVA was applied with time as a within-subjects factor and condition and sex as between-subjects factors to test the effect of the intervention. There were no significant main effects of time, condition or sex, or significant time × condition, time × sex or time × condition × sex interactions (for descriptive statistics see Appendix D, and for test statistics see Appendix E).

#### 3.2.4. Cognitive Flexibility

There was one outlier we had to exclude from the analysis; thus, the final sample consisted of 33 participants in the analysis regarding cognitive flexibility. We used a repeated-measures ANOVA again, with time as a within-subjects factor and condition and sex as between-subjects factors, to test the effect of the intervention. There were no significant main effects of time, condition or sex, and no significant time × condition, time × sex or time × condition × sex interactions (for descriptive statistics see Appendix D, and for test statistics see Appendix E).

### 3.3. Morning Cortisol Levels

#### 3.3.1. Pre-Test Differences

From the total sample (*n* = 51), for two children we did not succeed in collecting a sufficient amount of saliva (one in the control and one in the intervention group) on the pre-test; thus, they were excluded from all morning cortisol analyses. A further 16 children were missing from preschool (6 in the intervention and 10 in the control group) during the post-test week. In the case of one participant in the control group, the elementary school refused to participate, so they had to be excluded from analyses regarding follow-up assessment of cortisol. During data collection in September, the volume of saliva of another seven children was insufficient (5 in the intervention and 2 in the control group). Regarding the cortisol data in October, one child dropped out of the study (in the control group) due to absence from school, and the volume of saliva of five children was insufficient (4 in the intervention and 1 in the control group).

Because of the two-wave data collection (the summers of 2017 and 2018), we checked if there were any differences between the two years’ cortisol results. Significantly higher cortisol concentrations were measured at pre-test in 2018 (*M* = 0.106, *SD* = 0.047; *n* = 22) than in 2017 (*M* = 0.068, *SD* = 0.036; *n* = 27); *t*(47) = −3.24, *p* = 0.002) which is most likely due to measurement error. Because of this difference, we standardized the change in cortisol values from pre-test to post-test/follow-up in the two years and used these variables as the dependent variables in the ANOVAs.

In order to investigate the differences in standardized cortisol values at the pre-test, we ran univariate ANCOVAs in each subsample (participants included in the change from pre- to post-test, from pre-test to September and from pre-test to October cortisol analyses). We used standardized pre-test cortisol values as the dependent variable, condition and sex as fixed factors and the time of sampling as a covariant.

Regarding the data of the participants with pre- and post-test cortisol data (*n* = 32), there were no effects of condition or sampling time, as shown in Appendix C. Although not significant, there was a large-sized difference between boys and girls. In addition, a non-significant but large effect of interaction was found for condition and sex. Following up on this interaction, the difference between boys and girls was detectable only in the intervention group (*F*(1,16) = 7.69, *p* = 0.01, *η^2^* = 0.325) but not in the control group (*F* (1,11) = 0.01, *p* = 0.94, *η^2^* = 0.001). These possible differences were also tested in the case of those participants who could be included into the analyses regarding the follow-up assessment. In the case of those participants who were included in the pre-test to September cortisol analysis, there was no effect of condition, sex or sampling time and no significant interaction between condition and sex. Similarly, we tested these effects in the subsample included in the pre-test to October analysis. In this group, there was also no effect of condition, sex or sampling time and no significant condition × sex interaction (for descriptive statistics see Table 2, and the test results see Appendix C).

#### 3.3.2. The Effects of the Intervention

A univariate ANCOVA was applied to test the effects of condition on children’s change in baseline cortisol levels from pre- to post-test, with the standardized change in cortisol levels from pre- to post-test as a dependent variable, condition and sex as fixed factors and the two sampling times as covariates. One outlier had to be excluded for normal distribution. Levene’s test indicated unequal error variances (*F*(3,28) = 3.15, *p* = 0.04). There were no effects of condition, pre- or post-test sampling times, or a significant condition × sex interaction. Though not significant, there was a large effect of sex: boys’ cortisol levels decreased from pre- to post-test (*M* = −0.440, *SD* = 0.966), while girls’ cortisol levels increased (*M* = 0.134, *SD* = 0.866) (for the descriptive statistics and the test results, see Table 3).

To test the effect of the intervention after school entry, again, we conducted a univariate ANCOVA, with the standardized change scores in cortisol values from the pre-test in August to the first week of school in September as the dependent variable, condition and sex as fixed factors and the pre-test and follow-up sampling times as covariates. There was no main effect of the sampling times and no main effect of either condition or sex, but there was a significant condition × sex interaction (for the descriptive statistics and the test results, see Table 3).

**Figure 3:**
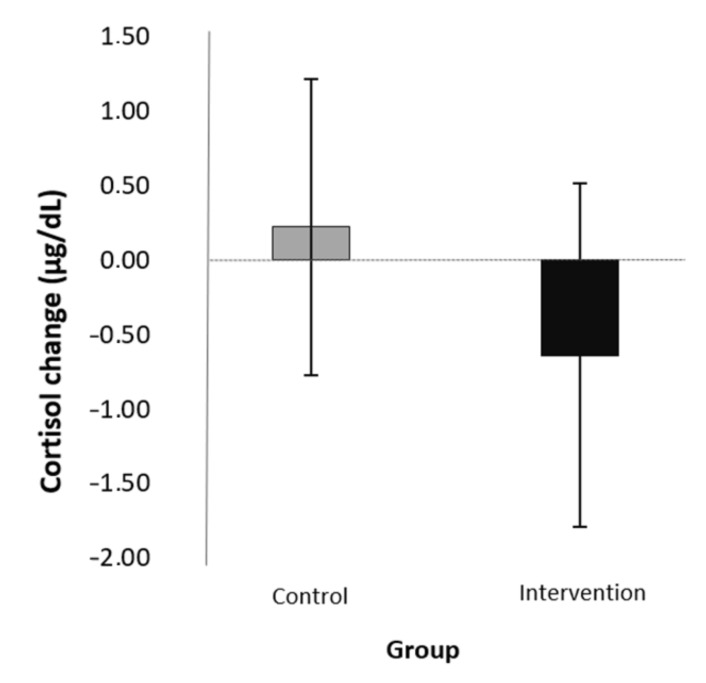
Effects of the intervention (mean of the change scores and SD) on morning cortisol levels from pre-test to September in boys.

**Figure 4:**
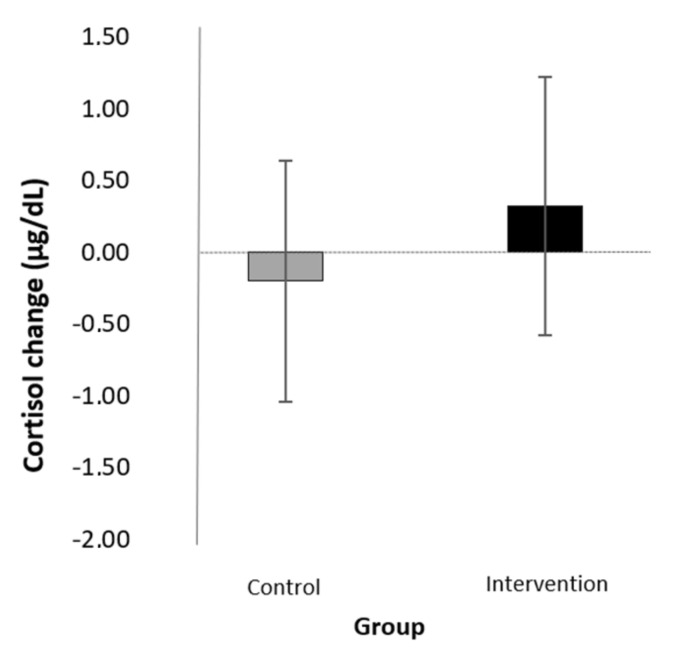
Effects of the intervention (mean of the change scores and SD) on morning cortisol levels from pre-test to September in girls.

In order to investigate the condition × sex interaction effect, we ran univariate ANCOVAs for boys and girls separately. We used standardized change scores in cortisol as the dependent variable and condition as a fixed factor. The two sampling times were used as covariates again. For the boys, there was no main effect of either the pre-test sampling times (*F*(1,14) = 0.02, *p* = 0.897, η^2^ = 0.001) or the follow-up sampling times (*F*(1,14) = 0.25, *p* = 0.625, η^2^ = 0.018). The main effect of condition was found to be large, though it did not reach significance (*F*(1) = 2.62, *p* = 0.128, η^2^ = 0.158). More specifically, boys’ cortisol levels decreased in the intervention and not in the control group (for standardized change scores, see Table 3 and Figure 3) For the girls, there was no main effect of the pre-test sampling times (*F*(1,19) = 0.17, *p* = 0.687, η^2^ = 0.009) but there was a significant main effect of the follow-up sampling times (*F*(1,19) = 6.50. *p* = 0.020, η^2^ = 0.255). The effect of the condition was small and not significant (*F*(1) = 0.29, *p* = 0.600, η^2^ = 0.015) (for standardized change scores in each group, see Table 3 and Figure 4).

In case of the second follow-up measure (the change from August to October), again, we used standardized change scores in cortisol values as the dependent variable, condition and sex as fixed factors and the pre-test and follow-up sampling times as covariates in the univariate ANCOVA. There were no main effects of either the pre-test cortisol sampling time, the follow-up cortisol sampling time, condition or sex. In addition, there was no condition × sex interaction (for standardized change scores in each group and statistics, see Table 3).

#### 3.3.3. Cortisol Reactivity (TSST-C)

To test the effect of the intervention on children’ cortisol reactivity, we ran a univariate ANCOVA, with the standardized scores of the AUCg during the stress test as the dependent variable, condition and sex as fixed factors and the time of the first cortisol sample as a covariate. There were no significant main effects of sampling time, condition or sex on total cortisol production (AUCg). In addition, there was no significant condition × sex interaction (for standardized cortisol values and statistics, see Appendix F).

To test the change in cortisol (AUCi) during the stress test, we had to exclude a soft outlier from the dataset to fulfil the assumption of normal distribution. We used the same model presented above, despite the fact that Levene’s test of equality of error variances was significant (*F* = 3.11, *p* = 0.045). There were no effects of condition, sex, or sampling time and no significant condition × sex interaction (for standardized cortisol values and statistics, see Appendix F).

## 4. Discussion

In the present pilot study, the feasibility and the effects of short mindfulness-based relaxation training sessions were tested on preschoolers’ short-term memory and executive function skills, on morning cortisol levels and cortisol reactivity after the intervention and on morning cortisol levels upon school entry. We assessed the effects of this intervention in the summer of 2017 and 2018 compared to a passive control group. Effects of the intervention on short-term memory, executive functioning and cortisol reactivity were tested before and after the intervention in the kindergarten, while morning cortisol levels were also assessed at follow-up times one week and one month after school entry. This study aimed to test whether these programs are feasible in kindergartens. We also aimed to confirm the positive effects of mindfulness-based relaxation training on children’s executive functions skills [7] and test whether these effect contribute to maintain optimal physiological stress levels in children. We chose a life event considered to be stressful, school entry [30], to investigate these questions and also to test whether such an intervention can contribute to maintain optimal stress levels at school entry.

Regarding the feasibility of the intervention program, we found that children were willing to participate and comply with the instructions at all the five sessions of the mindfulness-based relaxation program. No children dropped out of the study during the intervention phase unless they went on holiday with their family. Thus, such an intervention is a feasible option in the last year of preschool, at least in smaller groups like in the present study. Further studies should assess whether it is feasible in larger groups, such as a whole preschool classroom.

In contrast to the results of systematic reviews [5,7], our results showed no effects of the mindfulness-based relaxation program on children’s working memory or cognitive flexibility. Unfortunately, due to a floor effect, we could not test the effects on inhibitory control skills. The discrepancy in the findings in the present study and the previous literature might be due to the relatively short intervention that we applied (only five sessions long, while mindfulness-based interventions are usually conducted over eight sessions) and the pacing of the program, as the five sessions were conducted over only a week. Thus, a short and intense mindfulness and relaxation program might not be as effective in fostering children’s executive functioning as the interventions that were found effective in previous studies. For instance, the length of mindfulness interventions in a meta-analysis focusing on fostering children’s executive functioning ranged from eight to 25 sessions [7]. More specifically, the study that showed improved working memory capacities used an eight-session-long (one hour per session) program [56]. Another study that found better inhibitory control skills applied an 18-session program with 20 min sessions [57]. Finally, a third study showed improved cognitive flexibility [58], in which the Master Mind curriculum was used, which is a four-week-long program with 20 lessons. Additionally, the majority of the previous experiments that found positive effects in the aforementioned meta-analysis applied tests of inhibitory control [7], which we could not analyze. Future studies should assess the specific effects of mindfulness programs on the different components of children’s executive function skills. In addition, the possibility of insufficient statistical power due to the small sample size in the present study should not be excluded.

Similarly, there was no effect of the intervention on children’s short-term memory. Surprisingly, children’s performance was found to decrease from pre- to post-test, regardless of the condition. This finding is puzzling and might be due to children’s fatigue and decrease in interest, as there was only one week between the pre- and the post-test.

In order to assess children’s baseline physiological stress levels and cortisol reactivity, we applied an objective biomarker, salivary cortisol levels in the morning and during a TSST task. In contrast to what we expected, the intervention had no effects on children’s morning cortisol levels from pre- to post-test, that is, from the beginning to the end of August. However, we found a significant interaction between condition and sex in the change in cortisol levels from pre-test to the first follow-up, taken right after starting elementary school in the first week of September. That is, the mean of cortisol levels of boys in the mindfulness-based relaxation group decreased, while that of the control group did not; in fact, there was a tendency for an increase. This difference was large in magnitude based on the effect sizes; however, it did not reach the level of significance. These preliminary findings are in line with previous research. Sibinga and colleagues [43] also found a decrease in cortisol in response to mindfulness in a sample of boys from low-SES families. Regarding the effects of sex on the efficacy of mindfulness programs, the results of previous meta-analyses vary. While Sanada and colleagues [13] did not find a sex effect in studies regarding salivary cortisol levels in adult samples, Koncz et al. [14] showed that meditation interventions are more effective for reducing males’ blood cortisol. The present study is the first to our knowledge to raise attention to the role of sex in the efficacy of mindfulness-based programs in a child sample.

In summary, we believe that this finding highlights potential individual differences in children’s reaction to mindfulness-based interventions. Future research should further investigate children’s sex as a potential moderator of intervention efficacy.

The interaction effect between sex and condition faded for the second follow-up assessment in the first week of October. Thus, the mindfulness-based program applied in kindergarten in August had a different effect on the physiological stress levels of the two sexes several weeks later, right after school entry. The fact that this effect appeared on the assessment in September and not on the post-test or on the second follow-up in October might be explained by the previous finding that school entry may be a stressful life event [32], for which a stress reduction program could show a visible effect.

Finally, the authors did not find any effect of the intervention on cortisol reactivity. This is in contrast to previous results. Again, Creswell and colleagues [46] found an increase in reactivity after a three-session-long intervention, while trait mindfulness in Brown and colleagues’ [45] study was associated with lower stress reactivity. It is conceivable that the low sample size in the present study and a probable low statistical power resulted in the non-significant result, although it should be noted that effect sizes were moderate at best.

In summary, the results highlight the role of individual differences such as sex on intervention efficacy in the context of mindfulness-based programs. The program seemed to help prevent a rise in boys’ cortisol levels upon school entry. However, we found no effects on children’s executive function skills. It is possible that an intervention at the time of a stressful life event such as school entry is more helpful than a preventive measure, such as the one in the present study. Accordingly, the authors conducted a similar experiment, with the mindfulness program applied after school entry, and, interestingly, found no effects on cortisol levels but benefits on working memory capacity [59]. Further studies should focus on disadvantaged samples.

## 5. Limitations

First of all, it should be noted that the number of the participants was low in the present experiment, particularly regarding the post-test and follow-up data, so it may be worthwhile to repeat the experiment with a larger sample. Quite a few children dropped out of the present study, but most of them did so not because they refused to participate in the intervention or testing sessions but because the study was conducted in August, and the children went on holiday with their families. We could not include the data of further participants in the analyses due to technical problems, more specifically, we did not manage to collect sufficient amounts of saliva using the swabs. Second, data collection was implemented during two summers, and most likely due to measurement error, there was a significant difference between the cortisol values in the two cohorts; however, this difference was eliminated by standardizing the scores. More importantly, cortisol sampling could not be synchronized to waking time. Although we used sampling time as a covariate in the models, we could not completely rule out that this had an effect. In fact, there was a significant difference in sampling times between samples collected at school and at preschool, showing that samples were generally taken earlier in the school as compared to the preschool due to children arriving earlier in the morning in the former. Accordingly, we could not test whether school entry elevated children’s cortisol levels. Third, similarly to other studies assessing mindfulness-based programs, the intervention was very complex in the current study as it involved elements of mindfulness, relaxation and yoga. Thus, we cannot be certain which intervention elements are associated with the effects [60]. In addition, it was a short, five-session program presented in the context of a narrative story, while mindfulness-based programs are often longer, consisting of about eight sessions. In fact, Koncz et al. [14] found that longer meditation interventions are more effective in reducing cortisol levels. We have to emphasize, however, that such interventions must be adjusted to the needs and attention span of the target population and the possible circumstances, and such considerations drove our decision toward a brief intervention. Finally, the effect of the intervention was compared to a passive control condition, which raises the question whether any effects are specific to a mindfulness-based relaxation program or the result of non-specific treatment effects. It is also important to note that we sampled children from eight state preschools in average SES neighborhoods; thus, we suspect that the results can be generalized to the middle-SES population.

## 6. Conclusions

In summary, a short, mindfulness-based intervention relaxation training is feasible in the kindergarten setting. Additionally, while we failed to confirm previous results regarding the beneficial effects of mindfulness interventions for children’s executive function skills, we found a significant sex difference in the effectiveness of a short mindfulness-based relaxation training session applied right before school entry on preschoolers’ physiological stress around school entry, indicated by their morning cortisol levels. In fact, while for boys we found a large effect size (although not significant), there was no effect for girls. Finally, we did not find any effect on stress reactivity. Thus, the present study suggests that a mindfulness-based relaxation training program might have an effect for boys and not for girls for stress management. However promising our results are, further evidence is needed in order to confirm our finding.

## Figures and Tables

**Table 1:** Descriptive statistics of the control and intervention group.

	Control		Intervention	
	Sample Size	Mean Age, (Months) (SD)	Sample Size	Mean Age, (Months) (SD)
Boy	13	80.00 (3.62)	8	84.25 (7.13)
Girl	12	83.17 (5.46)	18	81.39 (5.57)

**Table 2:** Baseline (pre-test) values in control and intervention group for each outcome included in post-test or follow-up measures.

Sampling Included on Testing	Subgroups															
	Control		Intervention		Boy		Girl		Control				Intervention			
									Boy		Girl		Boy		Girl	
	Mean (*SD*)	*n*	Mean (*SD*)	*n*	Mean (*SD*)	*n*	Mean (*SD*)	*n*	Mean (*SD*)	*n*	Mean (*SD*)	*n*	Mean (*SD*)	*n*	Mean (*SD*)	*n*
**Cortisol ^a^**																
Pre–Post cortisol change	−0.009 (0.906)	13	−0.020 (1.078)	19	0.448 (1.021)	11	−0.259 (0.914)	21	0.015 (0.633)	5	−0.025 (1.084)	8	0.809 (1.191)	6	−0.402 (0.805)	13
Pre–September cortisol change	−0.023 (0.933)	21	0.024 (1.065)	20	0.273 (1.062)	18	−0.214 (0.891)	23	0.087 (0.831)	11	−0.144 (1.067)	10	0.564 (1.371)	7	−0.267 (0.770)	13
Pre–October cortisol change	−0.009 (0.938)	21	0.009 (1.058)	21	0.291 (1.070)	18	−0.218 (0.881)	24	0.102 (0.835)	11	−0.131 (1.072)	10	0.589 (1.384)	7	−0.281 (0.753)	14
**Cognitive functions ^b^**																
Digit span forward	5.33 (1.23)	15	5.16 (1.50)	19	5.46 (1.85)	13	5.10 (1.00)	21	5.86 (1.46)	7	4.88 (0.84)	8	5.00 (2.28)	6	5.23 (1.09)	13
Digit span backward	2.73 (1.16)	15	2.42 (1.12)	19	2.77 (1.24)	13	2.43 (1.08)	21	2.57 (1.51)	7	2.88 (0.84)	8	3.00 (0.89)	6	2.15 (1.14)	13
DCCS	7.29 (1.98)	14	7.37 (2.11)	19	6.92 (1.44)	12	7.57 (2.29)	21	6.83 (1.17)	6	7.63 (2.45)	8	7.00 (1.79)	6	7.54 (2.30)	13

Note: ^a^ Results are standardized values of changes in cortisol levels. ^b^ Achieved scores on each test. DCCS = Dimensional Change Card Sort.

**Table 3:** Standardized change scores and the effects of the intervention on cortisol levels.

Measurement	Standardized Change Scores								Results of Univariate ANCOVA																			
	Control				Intervention				Pre-Test Sampling Time				Post/Follow-Up Sampling Time				Condition				Sex				Condition × Sex			
	Boy		Girl		Boy		Girl																					
	Mean (*SD*)	*n*	Mean (*SD*)	*n*	Mean (*SD*)	*n*	Mean (*SD*)	*n*	*F*	*df*	*p*	*η^2^*	*F*	*df*	*p*	*η2*	*F*	*df*	*p*	*η^2^*	*F*	*df*	*p*	*η^2^*	*F*	*df*	*p*	*η^2^*
Pre-test–Post-test	−0.44 (0.20)	5	0.40 (1.19)	8	−0.44 (1.36)	6	−0.03 (0.59)	13	1.42	1,26	0.24	0.052	0.004	1,26	0.95	0	0.28	1,26	0.6	0.011	3.17	1,26	0.09	0.109	0.15	1,26	0.7	0.006
Pre-test–September	0.22 (0.99)	11	−0.20 (0.84)	10	−0.64 (1.15)	7	0.32 (0.90)	13	0.1	1,35	0.75	0.003	3.31	1,35	0.08	0.086	1.31	1,35	0.26	0.036	1.14	1,35	0.29	0.032	5.43	1,35	0.026	0.134
Pre-test–October	0.23 (0.96)	11	−0.054 (0.95)	10	−0.53 (1.15)	7	0.12 (0.95)	14	1.23	1,36	0.27	0.033	2.68	1,36	0.11	0.069	1.85	1,36	0.18	0.049	1	1,36	0.32	0.027	2.47	1,36	0.125	0.064

## Data Availability

The datasets analyzed for this study can be found in The Open Science Framework (https://osf.io/w4g6h/, accessed on 11 October 2020).

## Appendix A

**Table A1:** Possible differences in the time of cortisol sampling.

Sampling Included	Repeated-MEASURES ANOVA																							
	Time				Condition				Sex				Time × Condition				Time × Sex				Time × Condition × Sex			
	*F*	*df*	*p*	*η^2^*	*F*	*df*	*p*	*η^2^*	*F*	*df*	*p*	*η^2^*	*F*	*df*	*p*	*η^2^*	*F*	*df*	*p*	*η^2^*	*F*	*df*	*p*	*η^2_2_^*
Pre–Post cortisol change	2.27	1,29	0.14	0.072	0.44	1,29	0.51	0.015	0.61	1,29	0.44	0.021	0.34	1,29	0.57	0.012	1.20	1,29	0.28	0.039	11.54	1,29	0.23	0.050
Pre–September cortisol change	76.40	1,37	<0.001	0.674	1.69	1,37	0.20	0.044	0.11	1,37	0.74	0.003	0.01	1,37	0.91	<0.001	0.55	1,37	0.46	0.015	1.11	1,37	0.30	0.023
Pre–October cortisol change	77.30	1,38	<0.001	0.670	1.21	1,38	0.28	0.031	0.004	1,38	0.95	<0.001	0.001	1,38	0.97	<0.001	0.49	1,38	0.49	0.013	0.89	1,38	0.35	0.023

## Appendix B

**Table A2:** Schedule of the intervention.

Day 1	Day 2	Day 3	Day 4	Day 5
1. Breathing meditation Sea cotter cove 2. Sensory meditation: Focusing on sounds 3. Yoga Postures 4. Breathing meditation Sea cotter cove, continued	5. Muscle relaxation Angry octopus 6. Sensory meditation Touching snail shells 7. Sensory meditation Walking meditation	8. Breathing meditation Meet again 9. Sensory meditation Focusing on sounds 10. Sitting meditation Sitting still like a frog	11. Relaxation story Bubble riding 12. Sensory meditation Mindful eating 13. Short story Short summary of the bubble riding story 14. Sitting meditation A safe place	15. Introductory story Sitting meditation The pause button 16. Short storytelling 17. Yoga Postures 18. Short storytelling 19. Sitting meditation The conveyor belt of worries 20. A brief summary of what has been learned during the program

Sensory and breathing meditation tasks and story elements are based on the Hungarian translations of Lori Lite’s books, such as Sea Cotter Cove: A Relaxation Story [61], Angry Octopus: An Anger Management Story, introducing active progressive muscular relaxation and deep breathing [62] and Bubble Riding: A Relaxation Story, designed to teach children visualization techniques to increase creativity while lowering stress and anxiety levels [63]. Sensory meditation tasks are inspired by Susan Kaiser Greenland’s Mindful Games: Sharing Mindfulness and Meditation with Children, Teens, and Families [64].

Sound recordings of sitting meditations are the modified versions of the Hungarian version of Eline Snel’s book Sitting Still Like a Frog: Mindfulness Exercises for Kids (and Their Parents) [65].

Some of the yoga postures were based on the Hungarian version of Gilles Diederichs’ book Playful relaxation—35 relaxing games for children [66].

## Appendix C

**Table A3:** Group and sex differences at pre-test.

Sampling Included	Univariate ANOVA															
	Condition				Sex				Condition × Sex				Pre-Test Sampling Time			
	*F*	*df*	*p*	*η^2^*	*F*	*df*	*p*	*η^2^*	*F*	*df*	*p*	*η^2^*	*F*	*df*	*p*	*η^2^*
**Cortisol**																
Pre–Post cortisol change	0.24	1,27	0.63	0.009	3.43	1,27	0.08	0.113	3.43	1,27	0.08	0.113	1.66	1,27	0.21	0.058
Pre–September cortisol change	0.38	1,36	0.54	0.010	2.63	1,36	0.11	0.068	1.05	1,36	0.31	0.028	0.27	1,36	0.61	0.007
Pre–October cortisol change	0.33	1,37	0.57	0.009	2.97	1,37	0.09	0.074	1.18	1,37	0.28	0.031	0.21	1,37	0.65	0.006
**Cognitive functions**																
Digit span forward	0.25	1,30	0.62	0.008	0.57	1,30	0.46	0.019	1.49	1,30	0.23	0.047	-	-	-	-
Digit span backward	0.13	1,30	0.72	0.004	0.45	1,30	0.51	0.015	2.03	1,30	0.17	0.063	-	-	-	-
DCCS	0.003	1,29	0.96	0.000	0.75	1,29	0.39	0.025	0.03	1,29	0.87	0.001	-	-	-	-

Note: DCCS = Dimensional Change Card Sort.

## Appendix D

**Table A4:** Achieved digit span and DCCS scores of those participants who participated in both pre- and post-tests.

Test	Pre-Test								Post-Test							
	Control				Intervention				Control				Intervention			
	Boy		Girl		Boy		Girl		Boy		Girl		Boy		Girl	
	Mean (*SD*)	*n*	Mean (*SD*)	*n*	Mean (*SD*)	*n*	Mean (*SD*)	*n*	Mean (*SD*)	*n*	Mean (*SD*)	*n*	mean (*SD*)	*n*	Mean (*SD*)	*n*
Digit spanforward	5.86 (1.46)	7	4.88 (0.84)	8	5.00 (2.28)	6	5.23 (1.09)	13	5.14 (1.57)	7	4.63 (1.30)	8	4.17 (1.17)	6	5.00 (1.15)	13
Digit spanbackward	2.57 (1.51)	7	2.88 (0.84)	8	3.00 (0.89)	6	2.15 (1.14)	13	2.71 (0.49)	7	2.50 (2.00)	8	2.83 (0.75)	6	2.08 (1.12)	13
DCCS	6.83 (1.17)	6	7.63 (2.45)	8	7.00 (1.79)	6	7.57 (2.93)	13	6.50 (1.23)	6	7.88 (3.14)	8	7.17 (2.23)	6	7.85 (2.97)	13

Note: DCCS = Dimensional Change Card Sort.

## Appendix E

**Table A5:** Effects of the intervention on short-term memory, working memory and shifting skills.

Measurement	Repeated-Measures ANOVA																							
	Time				Condition				Sex				Time × Condition				Time × Sex				Time × Condition × Sex			
	*F*	*df*	*p*	*η^2^*	*F*	*df*	*p*	*η^2^*	*F*	*df*	*p*	*η^2^*	*F*	*df*	*p*	*η^2^*	*F*	*df*	*p*	*η^2^*	*F*	*df*	*p*	*η^2^*
Digit span forward	6.02	1,30	0.02	0.167	0.41	1,30	0.53	0.013	0.06	1,30	0.80	0.002	0.02	1,30	0.91	.000	1.67	1,30	0.21	0.053	0.03	1,30	0.87	0.001
Digit span backward	0.26	1,30	0.61	0.009	0.25	1,30	0.62	0.008	1.59	1,30	0.22	0.050	0.00	1,30	0.99	.000	0.21	1,30	0.65	0.007	0.43	1,30	0.52	0.014
DCCS	0.07	1,29	0.80	0.002	0.05	1,29	0.82	0.002	1.14	1,29	0.29	0.038	0.13	1,29	0.72	.005	0.23	1,29	0.64	0.008	0.08	1,29	0.77	0.003

Note: DCCS = Dimensional Change Card Sort.

## Appendix F

**Table A6:** Effects of the intervention, sex and sampling time on stress-induced cortisol responses.

Measurement	Standardized Cortisol Values								Univariate ANCOVA															
	Control				Intervention				Sampling Time				Condition				Sex				Condition × Sex			
	Boy		Girl		Boy		Girl																	
	Mean (*SD*)	*n*	Mean (*SD*)	*n*	Mean (*SD*)	*n*	Mean (*SD*)	*n*	*F*	*df*	*p*	*η^2^*	*F*	*df*	*p*	*η^2^*	*F*	*df*	*p*	*η^2^*	*F*	*df*	*p*	*η^2^*
AUCg	0.30 (0.86)	7	−0.14 (1.45)	7	0.057 (0.68)	6	−0.16 (0.90)	9	1.37	1,24	0.25	0.054	0.066	1,24	0.80	0.003	0.58	1,24	0.45	0.024	0.03	1,24	0.86	0.001
AUCi	0.31 (0.96)	7	−0.12 (0.41)	6	−0.01 (0.56	6	−0.39 (1.26)	9	0.31	1,23	0.58	0.013	0.73	1,23	0.40	0.031	1.32	1,23	0.26	0.054	0.01	1,23	0.91	0.001

Note: means = standardized cortisol values, AUCg = Area Under the Curve with respect to ground, AUCi = Area Under the Curve with respect to increase.
